# bla_VIM_ and bla_IMP_ Genes Detection in Isolates of Carbapenem Resistant P. *aeruginosa* of Hospitalized Patients in Two Hospitals in Iran

**Published:** 2017-10-01

**Authors:** Behrang Kazeminezhad, Arezoo Bostanmanesh Rad, Atoosa Gharib, Sara Zahedifard

**Affiliations:** 1 *Dept. of Pathology, Modarres Hospital, Shahid Beheshti University of Medical Sciences, Tehran, Iran*; 2 *Shahid Modarres Clinical research and Development center, Shahid Beheshti University of Medical Sciences, Tehran, Iran*; 3 *Dept. of Microbiology, Shahid Beheshti University of Medical Sciences, Tehran, Iran*

**Keywords:** bla_VIM_, bla_IMP_, Metallo-β-Lactamase, Pseudomonas aeruginosa

## Abstract

**Background & objective:**

Beta-lactam antibiotics resistance specifically Imipenem and Meropenem, the last choices of treatment, causes fatal events in patients with *P.aeruginosa* infection. The aim of this study was to detect the VIM and IMP of metallo-beta-lactamase genes in 103 isolates of *P. aeruginosa *in two Iranian hospitals.

**Methods:**

In this study, we evaluated the susceptibility of *P. aeruginosa* to a range of β-lactam antibiotics using disk diffusion method as a standard biochemical test. Combined disk test of Imipenem (IMP) and Imipenem plus Ethylenediaminetetraacetic acid (EDTA) was performed as a phenotypic method to find metallo-beta-lactamase producing isolates.Using conventional PCR method; we evaluated VIM and IMP of metallo-beta-lactamase (MBL) genes in 103 isolates of *P.aeruginosa*.

**Results:**

Twenty six (25.2%) out of 103 isolates were resistant to Imipenem and 26 (25.2%) to Meropenem. Among 26 Imipenem and Meropenem-resistant strains (25.2%), 19 cases (73.0%) were MBL producing. Using PCR method, we detected the bla_VIM _and bla_IMP _genes in 6 (5.8%) and 2(1.9%) of 19 MBL producing isolates, respectively.

**Conclusions:**

Evaluation of these carbepenemases genes improve epidemiologic researches and also, can be used as a diagnostic tool for discriminating between antibiotics resistant and sensitive strains of *P.aeruginosa* as well as follow-up the patients after treatment.

## Introduction

*P. aeruginosa* is known as one of the most important species of Pseudomonadaceae family and a common type of nosocomial pathogens with high mortality, mostly in immunocompromised patients(1, 2). The development of resistance to many antibiotics is the reason of high mortality rate particularly in healthcare (2). The most potent anti pseudomonal drugs are carbapenems including Meropenem (MEM) and Imipenem (IPM) that often administered as the last choice of treatment in patients infected by multi- β-lactam-resistant pseudomonas(3). Beta-lactam antibiotics is hydrolyzed by a various group of metallo-enzymes called Metallo-β-Lactamase (MBLs) because of inserting MBLs encoding genes in integrons and plasmids so, they have raised a serious problem in treatment of multi-resistant *P.aeruginosa* in hospitals (4). Important types of MBLs in Pseudomonas spp. include IMP, VIM, SPM, GIM, AIM, DIM, NDM and SIM type carbapenemases(5, 6). 

The suitable treatment and monitoring the spread of *P.aeruginosa* require evaluation of different factors. One of the most significant subjects is finding the beta lactamase producing isolates, specifically carbapenemase producing strains. The aim of this study was to find outbla_VIM_ and bla_IMP_ genes in 103 isolates of *P.aeruginosa* in a cohort of hospitalized patients from 2011 and 2012 in two Iranian hospital. 

## Materials and Methods

**Patients’ characteristics**

One hundred and three isolates from patients infected with *P. aeruginosa* in ShahidModarres and Milad hospitals from 2011 and 2012 were included in this study. Demographic and clinical data were recorded. 

**Microbial-resistance phenotypic evaluation**

 Antibiotic-resistant cultures of samples were identified and confirmed by standard biochemical tests. Antibiotic susceptibility test was done using gel disk diffusion method according to the Clinical and Laboratory Standards Institute (CLSI 2012). This test was done for antibiotics includingImipenem (10 µg), Meropenem (10 µg), Ticarcillin (75 µg), Ceftazidime (30 µ), Cefepime (30 µg) and Piperacillin (100 µg) (ROSCO Diagnostica, Taastrup, Denmark). A standard microbial suspension equal to 0.5 McFarland turbidity standards was prepared. The samples were cultured in the Mueller Hinton Agar medium using a sterile swab. 

The carbapenem-resistant isolates were more evaluated by combined disk test with Imipenem (IMP) and Imipenem plus Ethylenediaminetetraacetic acid (EDTA) as inhibitor of MBL_s _.The presence of a distorted inhibition zone around IMP-EDTA in comparison with IMP after overnight incubation was interpreted as a positive test result. An Imipenem disk (10 µg)and a prepared IMP-EDTA disk (containing 10 µg Imipenem and 750µg EDTA) were placed with an appropriate distance on a plate containing agar gel and carbapenem-resistant isolates. After 16-18 hours incubation of the plates at 37 ºC the zone diameter surround the disks were measured. A zone of equal to more than 7mm in the presence of 750 µg of EDTA compared to Imipenem disk alone was considered as a positive test for recognizing the MBL-producing resistant bacteria. 

**bla**_VIM_ **and bla**_IMP_ **gene Detection**

The DNA extraction for isolates of *P.aeruginosa* was performed using a DNA Extraction Kit (Invitek, Germany). 

**Polymerase chain reaction**

**Figure 1:**
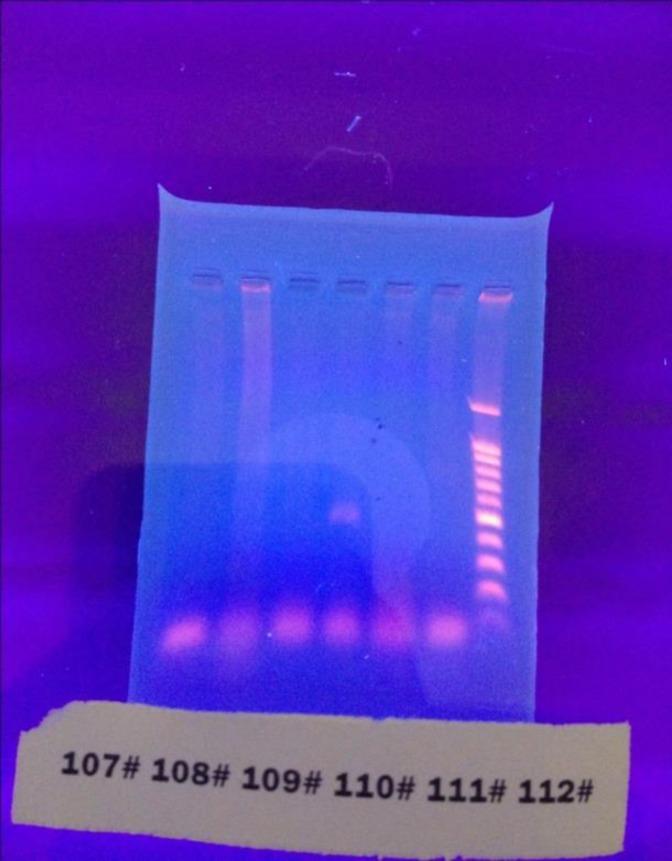
PCR for bla_VIM _gene. Gel electrophoresis showing positive and negative isolates. The DNA ladder in the right end of picture. The patient NO.110 has a band in 500 Kbp position which is the site of bla_VIM _gene. ( The DNA ladder is made of bands with 100, 200, 300, 400, 500, 600, 700, 800, 900, 1000, 1500 bp from bottom to the top

To perform PCR, 50ng DNA template was added to 25 μL of PCR mixture including 0.8 mM MgCl2, 200 μMdNTP, 10 pM of each primer containing: 

VIM Primer: GTT TGG TCG CAT ATC GCA AC AAT GCG CAG CAC CAG GAT AG, 

IMP primer: CTACCGCAGCAGAGTCTTTGC GAACAACCAGTTTTGCCTTACC (Metabion, Germany), and 0.5 U Taq DNA polymerase(Roche, Mannheim, Germany) (7). Thermocycler was used for DNA amplification (Techne, TC 512, UK). PCR procedure was as follows: 94°C for 2 minutes, 94°C for 1minute for 30 cycles, 55°C for 1 minute, 72°C for 1 minute, and 72°C for 10 minutes as final extension phase. The PCR product was mixed with 1 μL of loading buffer (×6) and subjected to gel electrophoresis on 1% agarose in a TAE buffer. After staining with ethidium bromide and visualizing amplicons by a Gel Documentation System, they were compared with a 100 bp ladder (7). (Fig 1)

## Results

In this study, the mean age of the patients was 49.98± 22.84 (Min=1y, Max=88y) with a gender distribution of 44.3% male and 55.7% female. Antibiotic-resistant cultures and subsequent molecular analysis were carried on samples of urine (n=60, 58.3%), respiratory tract (n=19, 18.4%), blood (n=6, 5.8%), phlegm (n=4, 3.9%) and the other sites (n=14, 13.6%). 

The frequency of resistance to different antibiotics was as follows: Imipenem (n=26, 25.2%), Meropenem (n=26, 25.2%), Ticarcillin (n=25, 24.3%), Ceftazidime (n=23, 22.3%), Cefepime (n=21, 20.6%) and Piperacillin (n=21, 20.4%). Among 103 isolates, 33% ( n=34) were resistant to at least one of the mentioned antibiotics. Sixteen patients (15.5%) with *P. aeruginosa *infection were multi-resistant to all above antibiotics. In our study from 26 Imipenem and Meropenem- resistant's isolates, 19 (73.0%) produce MBL with combined disk test. From 19 MBL producing isolates, 6 (31.5%) samples had bla_VIM _gene and 2 (10.5%) had bla_IMP _gene. Also, PCR for bla_VIM _and bla_IMP _was performed for 84 out of 103 isolates that are not resistant to carbapenemes and none of them show positivity for these genes. All of VIM-type carbapenemase positive samples (n=6, 100%) were isolated from urine. 

## Discussion

MBL-producing strains of *P.aeruginosa* in hospitalized patients are considered as a reason of spreading the nocosomial infections in healthcare settings and a major problem of physicians in the last decades (8, 9). An important problem of this organism is the development of drug resistance mechanisms. Some mechanism of resistance is including, efflux pumps, inactivation and modification of antibiotics, low permeability of the outer-membrane (10, 11). According to the reports, different strains of *P .aeruginosa* produce various amounts of MBL. It is so important concerning the association of serum MBL levels to the development of resistance to antibacterial antibiotics like imipenem, meropenem, anti pseudomonal cephalosporins and penicillin (12). In our study, 19(18.4%) of isolates were MBL producer, thus resistant to antibacterial antibiotics mostly imipenem. 

In this study, the most common sources of isolating *P.aeruginosa* infection were urine , respiratory tract (sputum) and blood, respectively, while Van der et al showed the high to low frequency of bacteria in respiratory tract, urine, blood, bone, soft tissue and abdominal samples, sequentially (13). In another study in South Africa, *P. aeruginosa* sources were indicated as blood, feces, bile and urine, in rank (14)which they are inconsistent with our findings. We also found six positive bla_VIM_ and two bla_IMP_ positive isolates among all samples. In contrast, Khosravi et al found no IMP carbapenemase-positive cases in MBL-producer *P.aeroginosa* (8). In a survey among burned patients, 57.9% of separated non-susceptible P. aeruginosa strains were MBL producers. All of these isolates show positivity for blaIMP-1 genes and none of them were positive for blaVIM genes (15). These findings are also in contrast with our findings.

According to the study of Hirakata in Japan, the mortality of patients infected by IMP-positive *P.aeruginosa* was higher than those contaminated by IMP-negative strains (16). Fortunately, the frequency of IMP producing isolates was lower than VIM in the present study. 

Yousefiet al showed that 17.31% of imipenem-resistant *P.aeruginosa*, had bla_VIM _gene in the samples taken from the patients in the northwest of Iran (17), which was somehow similar to our results. In another study reported by Shahcheraghiet al, they detected the bla_VIM _gene in 16 of 68 imipenem-resistant isolates (7)

The major difference between this study and the other similar researches is evaluating bla_VIM _gene and bla_IMP _genes in all of 103 pseudomonas strains in our study. In the present study, we could not find the bla_IMP_ and bla_VIM_ genes in antibiotic-susceptible isolates, indicating the functional activity of responsible agents to create a resistant phenotype in bacteria. 

Similar to our conclusion, MBL were identified in 10.8% of 415 isolates with a considerable raise in VIM prevalence in a cohort study in Korea (18). Cornaglia et al in Italy detected VIM-1 MBL gene in all 8 strains of carbapenem resistant *P. aeruginosa*(19). Higher percentage of VIM expression (35%) than our study has been also reported (13). Jacobson et alcould noticeably find VIM-2 gene in all 11 strains of drug resistant *P.aeruginosa* strains that they examined (14). 

Although there are less than 40% similarity between the IMP and VIM carbapenemase, their kinetic features for inactivating the beta-lactams, but not monobactam, are the same (12). In the worldwide, bla_VIM_ is a dominant gene which is related to the prevalence of nocosomial MBL-producing *P. aeruginosa*.

## Conclusion

In conclusion, the identification of carbapenemases in the antibiotic-resistant strains of *P.aeruginosa *may be a promising solution for the control of infection especially in hospitalized patients using the new treatment protocols. 
